# The Factors Affecting the Level of Women’s Awareness of Birth Preparedness and Complication Readiness in the Lake Zone, Tanzania: A Cross-sectional Study

**DOI:** 10.30476/IJCBNM.2020.86311.1336

**Published:** 2021-01

**Authors:** Ennegrace Nkya, Thecla W. Kohi

**Affiliations:** 1 Department of Community Nursing, School of Nursing, Muhimbili University of Health and Allied Sciences, Dar es Salaam, Tanzania; 2 Department of Public Health and Community Medicine, School of Nursing, St. Joseph University, Dar es Salaam, Tanzania

**Keywords:** District hospital, Health center, Pregnancy and postnatal

## Abstract

**Background:**

The concept of birth preparedness and complication readiness (BP/CR) has continued to generate interest in the last decade. Unfortunately, there is a paucity of published data regarding this subject in Tanzania and the Lake Zone in particular. This study aimed to determine the factors affecting the level of awareness of BP/CR among Tanzanian women in the Lake Zone.

**Methods:**

Between May and June 2016, a cross-sectional study on 737 postnatal or pregnant women was conducted in the Lake Zone Tanzania. A systematic random sampling technique was employed to select the study participants. A structured questionnaire adopted from the Johns Hopkins Program for International Education in Gynecology and Obstetrics (JHPIEGO) questionnaire was used to collect the data. Data analysis was carried out through SPSS (v.21) using statistical tests including descriptive Statistics, Chi-square tests and Multivariate logistic regression. A significance level of 5% was considered. The odds of the occurrence of events were assessed using Odds Ratios (OR) at a 95% confidence interval (CI).

**Results:**

The majority of women were multigravida (512=69.5%) with up to three living children (409=80%). Awareness of danger signs and BP/CR was low at 40% and 35%, respectively. Predictors of the level of awareness on BP/CR were multi-gravidity (P=0.04), awareness of at least three danger signs (P<0.001), and use of public transport (P=0.01).

**Conclusion:**

Low awareness of BP/CR in this study calls for strengthened efforts from policy-makers and healthcare providers to design effective programs geared towards educating women on the importance of BP/CR that will reduce the delays of seeking care, hence reducing maternal and neonatal deaths.

## INTRODUCTION

Birth preparedness is a comprehensive strategy used to improve the utilization of skilled providers at birth and a key intervention in reducing maternal mortality. ^1^ Birth preparedness encourages the women, households, and communities to make arrangements that facilitate quick decision-making during the three stages of childbirth and reduce delays in reaching care if or once a problem arises. ^2^ Globally, of about 300,000 mothers’ deaths in a year due to pregnancy complications and childbirth, about 99% occur in developing countries, with sub-Saharan Africa accounting for 56% and 29% in southern Asia. The rate of complications during childbirth is more than 15 times higher in the low- and middle-income countries compared to developed ones. ^3^

Data available in Tanzania shows that it is among the ten countries contributing to 56% of the global total maternal deaths. ^3^ The statistics in Tanzania are alarming as the maternal mortality rate (MMR) remained at 556 per 100,000 live births. ^4^ This is higher than the ratios of 454 per 100,000 live births and 432 per 100,000 live births reported by the Tanzania Demographic and Health Survey (TDHS) ^5^ and the United Nations ^6^ respectively. Tanzania was also one of the countries that did not attain its target goal of reducing the national MMR to 193 per 100,000 live births by December 31st, 2015. ^7^ It is estimated that, globally, in the absence of skilled obstetric care, 15% of all pregnant women will suffer from serious and long-term morbidities and disabilities. ^8^

Receiving care from a skilled provider during childbirth has been identified as an important intervention in reducing maternal and neonatal mortality rates (NMR). ^9^ However, the use of skilled providers in developing countries remains low. ^9 , 10^ Utilization of the principles and practices of BP/CR in low- and middle-income settings has the potential of reducing the existing high maternal and neonatal morbidity and mortality rates. BP/CR also encourages critical decision-making before the onset of labor. ^11^ Several studies have shown that delay in care-seeking is the most common indicator of delays in receiving quality care, ^12^ delays in reaching the primary point of skilled care, ^13^ and newborn mortality. ^14^ Thus, the promotion of BP/CR results in improved care-seeking during obstetric emergencies, leading to reduced morbidity and mortality related to childbirth. ^15 , 16^ A recent meta-analysis showed that BP/CR interventions could lead to 18% and 28% reduction in neonatal mortality risk and maternal mortality risk, respectively. ^17^

The World Health Organization’s model of Focused Antenatal Care (FANC) emphasizes counselling related to birth preparedness and, therefore, women are supposed to receive information and education on BP/CR during ANC visits. ^18^ In a rural Ugandan study that assessed the knowledge of obstetric danger signs and birth preparedness practices among women, the researchers found that women who attended the minimum recommendation of four visits for antenatal care were more likely to be prepared for assisted skilled birth. ^19^

Some studies have shown that the place of residence, type of occupation, educational level, family size, number of ANC attendance, knowledge of danger signs during pregnancy, labor and postnatal period, as well as gravida and parity are significantly associated with BP/CR. ^20 , 21^ A study by Mbalinda *et al*. ^22^ aimed to determine if knowledge of danger signs of pregnancy predicts the birth preparedness and found that only 36.5% of the respondents were regarded as knowledgeable on birth preparedness and complication readiness. A study in Nepal by Kaphle *et al*. on BP/CR found that 33.2% and 34.2% of women had knowledge and were prepared for all five BP/CR components, respectively. The factors influencing BP/CR were the women’s level of education, antenatal care attendance, and awareness of danger signs during pregnancy, delivery, and the postpartum period. In northwest Ethiopia, birth preparedness based on at least three components and knowledge of at least three danger signs during pregnancy, delivery, and the post-partum period was as low as 24% and 23%, respectively. Education level, history of stillbirth, antenatal care attendance during the last pregnancy, and male involvement during BP/CR counselling were found to be the factors associated with birth preparedness. ^23^ Other studies by David *et al*. and Urassa *et al*. that explored BP/CR among women in Mpwapwa District, Tanzania, showed that the majority (86.2%) of the women had decisions made on most of the components of BP/CR. However, only 8.7% of women arranged for a potential blood donor. ^24^

According to Kabakyenga *et al*., it was shown that 36.5% of women in Uganda had prior knowledge of deciding about where to deliver; of them, 56% had to consult their husbands and 8% consulted other relatives or friends. Women’s decision-making on the location of birth in consultation with spouse/friends/relatives and birth preparedness had a significant effect on seeking assistance from skilled birth attendants (SBAs) during delivery. ^25^

In Siddhart and colleagues’ study on BP/CR among women living in the slums of Indore City, India, it was found that 36.2% of the mothers did not identify a health facility for the delivery because they perceived that they would not face any complications while pregnant; hence, they planned for their deliveries to be conducted at home. ^26^ A qualitative study in Enugu State, Nigeria, found that low BP/CR was mainly linked with socio-demographic and economic characteristics, low knowledge of key danger signs, and negative attitudes on ANC services, coupled with the husband not committed to making preparations in advance for their wives’ childbirth needs. ^27^

According to 2010 TDHS data, only 27.3% of pregnant mothers are informed about pregnancy complications in Mwanza Region, ^5^ which is quite low when compared to the capital city, Dar es Salaam (79. 5%). 

Maternal mortality in Mwanza Region stands at 305 per 100,000 live births and the proportion of women giving birth at home comprises 54% of all births in the region, which is higher than the national figure of 50%. ^4 , 28^ Overall, Nyamagana District has been shown to have a relatively high maternal mortality ratio of 42%. Little research has been done to investigate the factors affecting awareness and uptake of BP/CR among women attending reproductive and child health clinics (RCHC) in Mwanza Region. 

This study aimed to determine the factors affecting the level of awareness of BP/CR among Tanzanian women in the Lake Zone.

## MATERIALS AND METHODS

This is a descriptive cross-sectional study that employed a quantitative approach. The design had been selected to get a snapshot of the level of awareness on BP/CR among pregnant women attending antenatal care clinics in Nyamagana District in Mwanza City. The study was carried out form 15^th^ May to 16^th^ June 2016.

Nyamagana District is one of the two districts in Mwanza City. It has a population of 363,452, of which 115, 337 are women of reproductive age. ^29^ It has 4 hospitals, 7 health centers, 17 dispensaries, 4 maternity homes, and 7 private clinics. The study sites were Nyamagana Hospital and Makongoro Health Centre (MHC), which were both facilities within the Nyamagana District. The two health facilities were purposefully selected as they have the highest referral of pregnant women in the District.

The study included the mothers who had given birth within two years before the study and brought their children to the clinics for growth monitoring at the two study sites. Also, pregnant women attending antenatal care clinics who had stayed in the district for more than 6 months and expected to give birth while still in the district were included in the study. A verbal and written informed consent to participate in the study was signed by the participants before the commencement of the study. Mothers with critically ill children were excluded from the study because it was thought that they may not be able to concentrate on the questions being asked and hence may provide unreliable information. 

The sample size of respondents was calculated using Epi-Info version 7.1.1.14. Clients were selected by systematic random sampling method from pregnant and postnatal mothers in the RCHC at Nyamagana hospital and MHC. It was estimated that 45-50 mothers of children under two years of age and pregnant women attend MHC and 35-50 refer to Nyamagana hospital on clinic days. Using a sample interval of every second woman to get time to interview women without keeping them waiting, were interviewed 22-25 women daily at Nyamagana hospital and 17-20 women at MHC. An average of 40 women were interviewed at both sites in a day and in two-week’s time a total of 737 respondents were interviewed. The first woman to be interviewed on each day was the first woman to come at the clinic.

Data were collected using a standar-dized, structured, interviewer-administered questionnaire. The questionnaire was adopted from the safe motherhood questionnaire developed by maternal and neonatal health program of JHPIEGO, affiliated to John Hopkins University, ^1^ with some modification based on the aim of the study. The questionnaire was pretested for validity and reliability. The items on the questionnaire were reviewed by the supervisors, colleagues and other experts for scrutiny, corrections, readability, clarity and comprehensiveness for face and content validity. The reliability of the items on the questionnaire was established using Cronbach’s alpha as a measure of internal consistency. The test has a reliability of 0.85 (F1-internal), 0.88 (F2-exaggerated) and 0.97 (F3-mediator). The BP/CR has been found to have excellent construct validity with a range of 0.85-0.95. To determine the reliability of the questionnaire, the validated version of the questionnaire was pretested with 5% of participants who agreed to take part in the pretest at Igoma health center. After pre-testing the questionnaire, Cronbatch’s Alpha was calculated using SPSS window version 21.0 to test the internal consistency (reliability) of the item and Cronbatch’s Alpha greater than 0.7 was considered as reliable. The Cronbach’s Alpha coefficient for the pretest questionnaires was 0.902 and 0.931 in the actual study implying that the items on the questionnaire correlate to each other. 

The questionnaire consists of five parts with a total of 42 questions. Part one of questionnaires gathered socio-demographic information of the respondents (12 questions). Part two was developed to collect information regarding obstetric characteristics of the respondents (6 questions). Part three was structured to capture information regarding the knowledge of danger signs and birth preparedness (7 questions). Part four gathered information regarding attitude towards BP/CR (8 questions) and Part five gathered information on personal experience with the last pregnancy (9 questions). The questionnaire was translated into Kiswahili language (the primary language spoken by most of the study participants) by a linguist proficient in both English and Kiswahili languages. The translated questionnaire was then reviewed by a third person who was also proficient in both languages. The questionnaire, originally written in English, was translated into local language (Kiswahili) and back to English in order to ensure that the translated version gives the proper meaning. The questionnaire was administered by two assistants (one at each site) who were trained nurses and who were also fluent in the local dialects, including Kiswahili. The assistants participated in specialized training for two days before piloting the tool. The questionnaire was pretested at Igoma health center on 5% of the sample size prior to the real data collection, which was excluded from the final study. Questionnaires were administered at the exit after receiving the health services in a special room set aside specifically for questionnaire administration at each site. This was done to allow the women to receive the services they came for first and to provide privacy during the data collection. Once the data were collected, each questionnaire was checked for completeness.

Assessment of the level of awareness of danger signs: If the respondent mentioned a total of five danger signs in all three phases with at least one in each phase, that is, one danger sign during pregnancy (vaginal bleeding, severe headache, blurred vision, swollen hands/face, high fever, convulsions, loss of consciousness, water breaks without labor, accelerated/reduced fetal movements, and tiredness/breathlessness); delivery (severe bleeding, severe headache, loss of consciousness, convulsions, high fever, labour lasting &gt;12 hours, and placenta not delivered 30 minutes after baby); and post-partum period (severe bleeding, severe headache, blurred vision, convulsions, swollen hands/face, high fever, loss of consciousness, difficulty in breathing, and severe weakness) plus at least two more danger signs from any of the three stages of childbirth, this respondent was considered to have good knowledge; otherwise, it was regarded as one with poor knowledge. ^11 , 20^

*Assessment of the level of awareness of BP/CR:* The respondent was considered to have awareness of BP/CR if she mentioned at least 3 components among five basic components of BP/CR, i.e. out of 5 options given (preparing for a place to give birth, identifying transport in case of emergency, identifying skilled attendant, saving money, and identifying a blood donor). Participants who had planned or had an intention to follow at least 4 of the five basic steps of BP/CR were considered well prepared for childbirth; otherwise, they were regarded as unprepared.

Recall and interviewer bias was the potential source of bias in this study. To minimize such a bias-, we used a standardized, structured, interviewer administered questionnaire adopted from JHPIEGO to maximize the accuracy and completeness. The questionnaire consisted of closed end, easy to understand questions with appropriate response options. Also, to minimize the recall bias in this study, only mothers who had given birth within the two years before the study and brought their children to the clinics for growth monitoring at the two study sites were involved.

Statistical data analysis was done using SPSS , Version 21. Categorical data were summarized using frequency distributions and the charts were used to enhance the visibility. Numerical data were summarized using descriptive statistics. Comparisons of categorical data were done using Chi-square testing attested at a statistical significance of P&lt;0.05. The odds of occurrence of events were assessed using Odds Ratios (OR) at a 95% confidence interval (CI). Mean differences for the continuous data were tested using independent samples t-test. Study variables that were found to be statistically significant in univariate analysis were subjected to multivariate logistic regression analysis to determine the predictor variables that predict BP/CR.

Ethical clearance was sought and obtained from the Muhimbili University of Health and Allied Sciences (MUHAS), Directorate of Research and Publications (Ref.No.MU/PGS/SAEC/Vol.IX). Permission to conduct the study was sought from the Regional Administrative Secretary (RAS) through the Regional Medical Officer (RMO) and Medical Officer in-charge (MO i/c) of the two health facilities selected in Nyamagana. Informed consent (verbal and written) was obtained from the respondents by reading to them the consent instructions, assuring them that their identity and the information they provided was kept confidential by having their names concealed by using codes. In participants younger than 18 years of age, informed consent was sought from parents or relatives. To minimize dishonesty, complete information was given to the participants by explaining the rationale of the research. The respondents were informed that participation in the study was entirely voluntary and they had the right to withdraw at any stage of the interviewing process with no negative impact on their future access to services at the health facility. The participants were protected following the four principals of Non maleficent, No harm to the client, Autonomy and Practicing justice.

## RESULTS

A total of 737 respondents were enrolled in the study, with a response rate of 98.4%. Their age ranged from 15 to 50 years with a mean±SD age of 26.2±5.9 years. 455 women (61.7%) were aged between 20 and 29 years. Table 1 shows the socio-demographic characteristics of the respondents. 

**Table 1:** Sociodemographic characteristics of the respondents

Variable	No. (%)
Health facility	
Makongoro	371 (50.3)
Nyamagana	366 (49.7)
Age (years)	
Under 20	76 (10.3)
20–29	455 (61.7)
30–39	189 (25.6)
40–50	17 (2.3)
Marital status	
Married/co-habiting	624 (84.7)
Single	78 (10.6)
Divorced/separated	28 (3.8)
Widowed	7 (0.9)
Education level	
Non-formal	36 (4.9)
Primary–incomplete	45 (6.1)
Primary–complete	466 (63.2)
Secondary	147 (19.9)
Post-secondary	43 (5.8)
Occupation	
House work	297 (40.3)
Peasant	40 (5.4)
Business–petty	352 (47.8)
Civil servant	16 (2.2)
Employee - private sector	29 (3.9)
Other (student)	3 (0.4)
Religion	
Christian	523 (71.0)
Muslim	207 (28.1)
Other (Pagan)	7 (0.9)
Average monthly income (TSh) (n=727)	
Less than 50,000	355 (48.8)
50,000-99,999	107 (14.7)
100,000-199,999	178 (24.5)
200,000-499.999	70 (9.6)
500,000-999,999	11 (1.5)
1,000,000 or higher	6 (0.8)
Family size	
1–2	69 (9.4)
3–5	507 (68.8)
More than 5	161 (21.8)
Time taken to reach the health facility (minutes)	
Mean (SD, Range)	35.1 (26.3, 2-180)
0–29	274 (37.2)
30–59	286 (38.8)
60 or more	177 (24.0)
Means of transport to reach health facility	
Public bus	293 (39.9)
Motorcycle (bodaboda)/bicycle	59 (8.0)
Private transport	15 (2.0)
Walking (on foot)	366 (49.7)
Other means (ferry/panton)	4 (0.5)

TSh: Tanzanian shilling (currency)

Regarding the obstetric characteristics of the respondents, 512 (69.5%) were multigravida. Of these 426 (83.2%) had exprienced up to four pregnancies with 3.2±1.4 pregnancies (Table 2).

**Table 2:** Obstetric characteristics of the respondents

Variable	No. (%)
Gravidity (N=737)	
Prime gravid	225 (30.5)
Multigravida	512 (69.5)
Current pregnancy status (N=737)	
Currently pregnant	204 (27.7)
Already given birth	533 (72.3)
Number of multigravida (N=512)	
Up to 4	426 (83.2)
More than 4	86 (16.8)
Number of times given birth (N=512)	
Up to 3	392 (76.6)
More than 3	120 (23.4)
Occurrence of stillbirths (N=512)	
Yes	126 (24.6)
No	386 (75.4)
Number of living children (N=512)	
Up to 3	409 (79.9)
More than 3	103 (20.1)
Number of antenatal care visits (N=737)	
Less than 4	260 (35.3)
4 or more	477 (64.7)
Gestation age at first ANC visit (weeks) (N=737)	
Up to 16	444 (60.2)
More than 16	293 (39.8)

**Figure 1:**
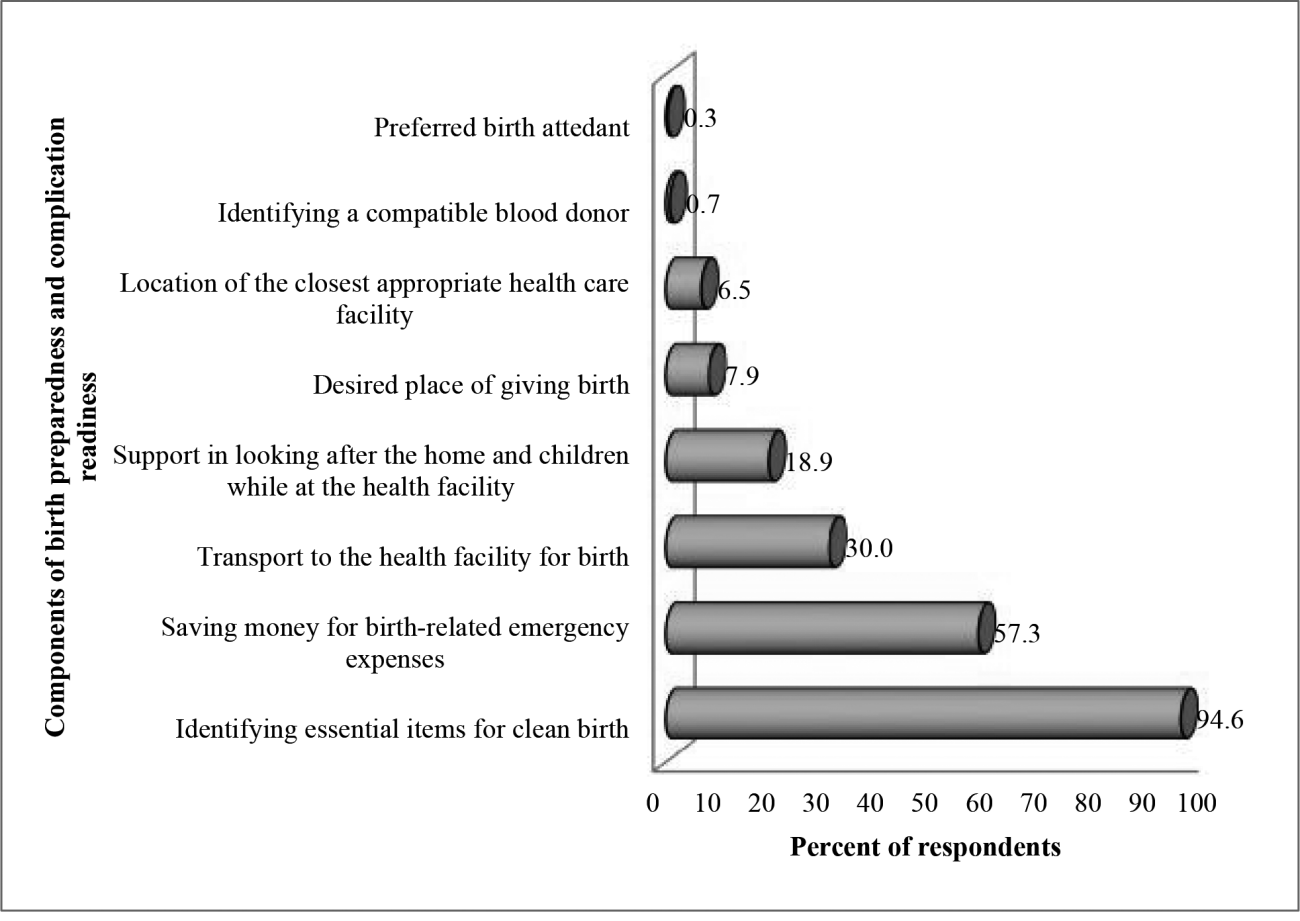
Women’s awareness of components of birth preparedness and complication readiness

Of the737 respondents, only 261 (35.4%) mentioned at least three of the five main components of BP/CR; hence, awareness was low. The mean score of awareness on BP/CR was 2.2±1.2. Out of 737 respondents, only 295 (40.0%) were able to mention at least five danger signs during the three phases (pregnancy, labour and childbirth, and postpartum), signifying low awareness of danger signs. Study respondents were asked if they had ever heard about BP/CR. Among 737 respondents, 721 (97.8%) stated they had heard about BP/CR. The main source of information about BP/CR was from health professionals during antenatal care clinic attendance (89.7%). The distribution of known components of BP/CR is shown in Figure 1; the most commonly mentioned components were identifying essential items for a clean birth (69794.6%) and saving money for birth-related emergency expenses (422=57.3%).

**Figure 2:**
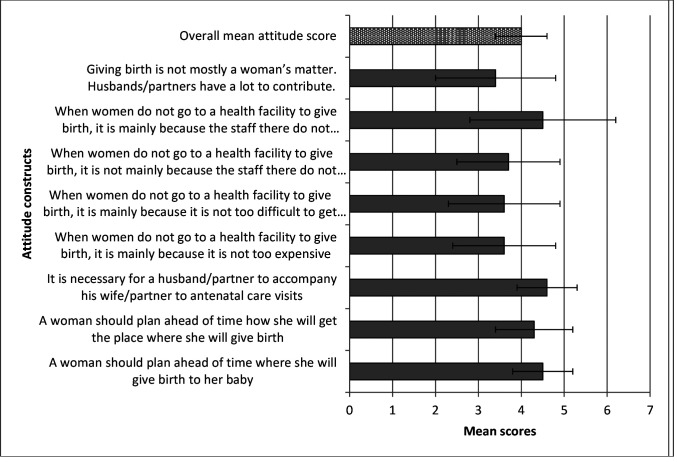
Mean scores on the constructs of women’s attitude towards birth preparedness and complication readiness

The overall mean of birth preparedness and attitude score of women was 4.0±0.6, indicating the positive attitude of women towards BP/CR. Figure 2 shows the distribution of mean attitude scores. The proportion of women demonstrating positive attitudes towards BP/CR was 629 (85.3%).

Table 3 shows the relationship between socio-demographic characteristics of the participants and the level of awareness of BP/CR according to univariate analysis. Obstetric factors influencing the level of awareness of BP/CR according to univariate analysis are shown in Table 4. 

**Table 3:** Relationship between socio-demographic characteristics of the participants and level of awareness of birth preparedness and complication readiness according to univariate analysis

Variable	Level of awareness of BP/CR		OR (95% CI)	P value*
	High	Low		
	No (%)	No (%)		
Health facility				
Makongoro	118 (31.8)	253 (68.2)		
Nyamagana	143 (39.1)	223 (60.9)	0.7 (0.5-1.9)	0.23
Age (years)				
Under 30	178 (33.5)	353 (66.5)		
30–50	83 (40.3)	123 (59.7)	0.7 (0.5-1.0)	0.08
Marital status				
Never married	22 (28.2)	56 (71.8)		
Ever married	239 (36.3)	420 (63.7)	0.7 (0.4-1.2)	0.15
Education level				
Up to primary	177 (32.4)	370 (67.6)		
Above primary	84 (44.2)	106 (55.8)	0.6 (0.4-0.8)	˂0.001
Occupation				
Formally employed	20 (44.4)	25 (55.6)		
Not formally employed	241 (34.8)	451 (65.2)	1.5 (0.8-2.8)	0.19
Religion				
Christian	181 (34.6)	342 (65.4)		
Non-Christian	80 (37.4)	134 (62.6)	0.9 (0.6-1.2)	0.47
Average monthly income (TShs)				
Less than 200,000	224 (35.0)	416 (65.0)		
200,000 or more	34 (39.1)	53 (60.9)	0.8 (0.5-1.4)	0.45
Family size				
Up to 5	207 (35.9)	369 (64.1)		
More than 5	54 (33.5)	107 (66.5)	1.1 (0.8-1.6)	0.57
Time taken to reach health facility (minutes)				
Less than 60	211 (37.7)	349 (62.3)		
60 or more	50 (28.2)	127 (71.8)	1.5 (1.1-2.2)	0.02
Means of transport to health facility				
Public transport/ferry	124 (41.8)	173 (58.2)		
Non-public/walking	137 (31.1)	303 (68.9)	1.6 (1.2-2.2)	˂0.001

CI: Confidence interval; OR: Odds ratio;

* Pearson Chi Square

**Table 4:** Relationship between obstetric characteristics of the participants and their level of awareness of birth preparedness and complication readiness according to univariate analysis

Variable	Level of awareness of BP/CR		OR (95% CI)	P value*
	High	Low		
	No (%)	No (%)		
Gravida (n=737)				
First (prime gravida)	63 (28.0)	162 (72.0)		
Multi gravid	198 (38.7)	314 (61.3)	0.6 (0.4-0.9)	˂0.001
Gravida (n=512)				
2–4	166 (39.0)	260 (61.0)		
More than 4	32 (37.2)	54 (62.8)	1.1 (0.7-1.7)	0.76
Pregnancy status at study time (n=737)				
Currently pregnant	91 (44.6)	113 (55.4)		
Given birth within last 24 months	170 (31.9)	363 (68.1)	1.7 (1.2-2.4)	˂0.001
Parity (n=512)				
1–3	147 (37.5)	245 (62.5)		
More than 3	51 (42.5)	69 (57.5)	0.8 (0.5-1.2)	0.32
Occurrence of stillbirth(s) (n=512)				
Yes	52 (41.3)	74 (58.7)		
No	146 (37.8)	240 (62.2)	1.2 (0.8-1.7)	0.49
Number of living children (n=512)				
0–3	153 (37.4)	256 (62.6)		
More than 3	44 (42.7)	59 (57.3)	0.8 (0.5-1.2)	0.32
Number of antenatal care visits (n=737)				
Up to 3	102 (39.2)	158 (60.8)		
44 or more	159 (33.3)	318 (66.7)	1.3 (0.9-1.8)	0.11
Knowledge on danger signs (n=737)				
Adequate	171 (58.0)	124 (42.0)		
Inadequate	90 (20.4)	352 (79.6)	5.4 (3.9-7.5)	<0.001
Attitude on BP/CR (n=737)				
Positive	233 (37.0)	396 (63.0)		
Negative	28 (25.9)	80 (74.1)	1.7 (1.1-2.7)	0.02

CI: Confidence interval; OR: Odds ratio;

* Pearson Chi Square

According to multivariate logistic analysis, multi-gravidity (P=0.04), awareness of at least three danger signs (P<0.001), and using public means of transport (P=0.01) were the main predictors of the level of awareness of BP/CR, as shown in Table 5.

**Table 5:** Birth preparedness and complication readiness predicting variables according to multivariate logistic analysis

Variables	*P value	OR	95% C.I.	
			Lower	Upper
Education level (up to primary)	0.05	0.68	0.46	1.00
Time taken to reach health facility (less than 60 minutes)	0.23	1.28	0.85	1.92
Means of transport (public or ferry)	0.01	1.51	1.07	2.12
Gravidity (prime gravida)	0.04	0.67	0.45	0.99
Pregnancy status at time of study (pregnant)	0.05	1.43	0.99	2.06
Level of knowledge on danger signs (adequate)	<0.001	0.21	0.15	0.29
Attitude towards BP/CR (positive)	0.17	1.42	0.85	2.36

CI: Confidence interval; OR: Odds ratio;

* multivariate logistic regression analysis

## DISCUSSION

The results of this study showed that the level of awareness of BP/CR among women attending RCHC in Nyamagana District was unacceptably low (35.4%), a figure which is similar to 36.5% that was reported by Mbalinda *et al*. ^22^ in Uganda. A high level of awareness of BP/CR has been reported in other studies in sub-Saharan Africa, 70.5% in Kenya ^30^ and 74.3% in northern Ghana. ^31^ The level of awareness of BP/CR in our study was high compared to 24.1% and 23.3% that was reported in northwest Ethiopia ^23^ and in southwest Ethiopia, ^3^ respectively. This difference in the level of awareness of BP/CR in these studies could be attributed to variations in the study populations, the definition of the level of awareness/knowledge of BP/CR, and study settings (rural vs. urban). While most of these studies either included postnatal women who had given birth to their babies in the last 12 months or women in their third trimester, our study included both postnatal and pregnant women irrespective of the trimester. Interestingly, one study32 also included the expected date of delivery as a component of BP/CR.

The finding that the main source of information on BP/CR was provided by health professionals during antenatal care clinic attendance is consistent with other studies in Ethiopia. ^3 , 11^ Although the majority of the study participants (97.8%) in this study stated that they had heard about BP/CR from health professionals during antenatal care clinic attendance, most of them had inadequate knowledge about BP/CR. The low level of awareness of BP/CR among respondents in this study could be attributed to insufficient information provided by the health providers in the communication of the message on BP/CR.

In agreement with other studies in developing countries, ^2 , 12 , 13^ the most recalled childbirth preparation activities among pregnant women in the present study were preparing items to be used during childbirth and saving money for birth-related emergency expenses, which may be explained by the fact that both women know that money is required to facilitate referral in the case of complications. Studies on birth preparedness in Ethiopia, ^3^ India, ^26^ and Uganda ^19^ have reported saving money for birth-related emergency expenses. Other studies conducted in Burkina Faso ^17^ and in Ethiopia ^11^ found that most women had identified SBAs and health facility as the main birth preparedness practices, respectively. 

It is well known that awareness of danger signs in pregnancy is an important step in reducing delays in seeking care. ^21 , 32^ Studies conducted among women in developing countries indicate low levels of awareness of obstetric danger signs during pregnancy, delivery, and postpartum. ^20 - 26 , 31 - 33^ The low awareness of danger signs coupled with lack of preparedness contributes to the delay in seeking skilled care henceforth, leading to high levels of maternal mortality and morbidity. In this study, only 40.0% of the respondents were able to mention at least five danger signs during the three phases (pregnancy, delivery and postpartum), signifying low awareness of danger signs. In a study conducted in rural Uganda to assess the knowledge of obstetric danger signs and birth preparedness practices among women found that only 18.7% could mention at least 3 key danger signs in all the three periods. ^20^ Kuganab-Lem, *et al*. in a study carried out in rural Ghana among postpartum women found that 79.0% of the respondents were aware of obstetric danger signs. ^33^ The low level of awareness of danger signs in our study may indicate that the key danger signs are not emphasized during antenatal care, as our study showed that the majority of the respondents (64.7%) had attended at least four antenatal care visits during their last pregnancy. Improving knowledge about obstetric danger signs and promoting birth preparedness practices are the strategies which aim at enhancing utilization of skilled care in low-income countries. 

In this study, more than 85% of the participants exhibited positive attitudes towards BP/CR. This is higher than the proportion of women with favorable attitudes (61.0%) reported by Debelew *et al*. in southwest Ethiopia. ^3^ The favorable attitude in our study could have been attributed to the education that pregnant women had received during antenatal care visits as evidenced by the fact that approximately 98% of women were aware of BP/CR. Most of the information received regarding BP/CR was from health care workers during antenatal care visits.

In the present study, significant factors associated with the level of awareness of BP/CR were multi-gravidity, awareness of at least three danger signs and using public means of transport. The high level of awareness of BP/CR among multi-gravid women in this study is in agreement with the findings of Ethiopian and Indian studies which revealed that parity was a predictor of BP/CR, attributing this to the previous experience of health care service utilization and increased awareness about obstetric complications from the previous pregnancy. ^24 , 27^ The finding that the high level of awareness of at least three obstetric danger signs in our study was a predictor of BP/CR agrees with the results of other studies. ^18 , 22^ It is well established that awareness of key danger signs encourages the women and their families to prepare for emergencies during the process of childbirth, and hence increases their level of awareness concerning BP/CR. Although education level was not significantly associated with BP/CR on multivariate logistic regression analysis, several studies have found that the level of education was the best predictor of awareness of BP/CR. ^2 , 25 , 32 - 34^ It could be due to the fact that women who have attained high levels of education are able to adhere to counseling provided at ANC and better understand the health messages acquired from various sources. Every woman should be informed of the likelihood of complications during pregnancy, childbirth/labor, and the postnatal periods. Information should be availed to community members, including women and their spouses about danger signs. Interventions targeting improvement of maternal health need to consider the quality of antenatal care, together with the quality of information offered to pregnant women and their spouses. Knowledge of the key danger signs needs to be given priority as it prepares the women and their families for timely and appropriate decision making in the case of complications, whereas birth preparedness offers readiness to reach health facilities for normal or complicated childbirth.

The strength of this study is that the questionnaire was an adapted version of JHPIEGO and was used by a well-trained research assistant. However, the potential limitations of this study were the fact that the study was conducted in only two sites of Mwanza City. This limits the generalizability to other areas of the country due to the limited variability of the study population and study sites. Since the sample was picked from RCHC, there existed a risk of missing those who had not attended RCH, thus resulting in weaker representativeness of the study population. Also, the answers given by women were self-reported and might be influenced by their families, resulting in a bias. Since this study employed a cross-sectional study design, the cause-effect relationships between variables, therefore, cannot be shown. Also, this study included only women attending RCH clinic. Men (husbands) were not involved in this study. A study involving the husbands to obtain their views on the factors affecting the uptake of BP/CR would improve the validity of the findings as well.

## CONCLUSION

The findings of the study demonstrated that the level of awareness of BP/CR among women attending RCHC in Nyamagana District was unacceptably low. Significant factors responsible for the low level of awareness of BP/CR birth were multi-gravidity, awareness of at least three danger signs and using public means of transport. Attitude towards BP/CR is high and is one of the factors influencing the level of awareness of BP/CR, though not a predictor. The low awareness of BP/CR in this study calls for strengthened efforts from policy-makers and healthcare providers to design effective programs geared towards educating women on the importance of BP/CR that will reduce the delays of seeking care and hence reduce maternal and neonatal deaths. 
